# Wnt activation followed by Notch inhibition promotes mitotic hair cell regeneration in the postnatal mouse cochlea

**DOI:** 10.18632/oncotarget.11479

**Published:** 2016-08-22

**Authors:** Wenli Ni, Shan Zeng, Wenyan Li, Yan Chen, Shasha Zhang, Mingliang Tang, Shan Sun, Renjie Chai, Huawei Li

**Affiliations:** ^1^ Otorhinolaryngology Department of The Affiliated Eye and ENT Hospital, State Key Laboratory of Medical Neurobiology, Fudan University, Shanghai, PR China; ^2^ Institutes of Biomedical Sciences, Fudan University, Shanghai, PR China; ^3^ Central Laboratory, Affiliated Eye and ENT Hospital of Fudan University, Shanghai, PR China; ^4^ Key Laboratory of Hearing Medicine of The National Health and Family Planning Commission, Shanghai, PR China; ^5^ Key Laboratory for Developmental Genes and Human Disease, Ministry of Education, Institute of Life Sciences, Southeast University, Nanjing, PR China; ^6^ Co-Innovation Center of Neuroregeneration, Nantong University, Nantong, PR China

**Keywords:** Wnt, Notch, proliferation, regeneration, lineage tracing

## Abstract

Hair cell (HC) loss is the main cause of permanent hearing loss in mammals. Previous studies have reported that in neonatal mice cochleae, Wnt activation promotes supporting cell (SC) proliferation and Notch inhibition promotes the trans-differentiation of SCs into HCs. However, Wnt activation alone fails to regenerate significant amounts of new HCs, Notch inhibition alone regenerates the HCs at the cost of exhausting the SC population, which leads to the death of the newly regenerated HCs. Mitotic HC regeneration might preserve the SC number while regenerating the HCs, which could be a better approach for long-term HC regeneration. We present a two-step gene manipulation, Wnt activation followed by Notch inhibition, to accomplish mitotic regeneration of HCs while partially preserving the SC number. We show that Wnt activation followed by Notch inhibition strongly promotes the mitotic regeneration of new HCs in both normal and neomycin-damaged cochleae while partially preserving the SC number. Lineage tracing shows that the majority of the mitotically regenerated HCs are derived specifically from the Lgr5^+^ progenitors with or without HC damage. Our findings suggest that the co-regulation of Wnt and Notch signaling might provide a better approach to mitotically regenerate HCs from Lgr5^+^ progenitor cells.

## INTRODUCTION

Hearing loss is considered the most frequent sensory deficit in human populations, and it occurs at all ages worldwide [1]. Sensorineural hearing loss is the most common type of hearing loss, and it can be caused by various insults such as acoustic trauma, ear and brain tumors, aging, noise exposure, ototoxic medications, and chemicals. Sensorineural hearing loss cannot currently be medically treated [2] because of the irreversible death of hair cells (HCs) in the organ of Corti. Unlike birds or fish, effective HC regeneration has not been observed in the mature mammalian cochlea. Within the past few years, however, exciting animal research on genetic manipulation, gene therapy, and stem cell transplantation as well as new pharmaceutical agents have suggested that hearing loss might eventually be curable in the future.

In the mouse inner ear, supporting cells (SCs) have been shown to be a reliable source for regenerating HCs after damage in both the cochlea and the utricle [3–10]. Previous studies have demonstrated that HC regeneration occurs by two mechanisms in neonatal cochlea: mitotic regeneration, in which a SC reenters the cell cycle and then several days later one or both daughter cells changes fate to become a HC; and direct trans-differentiation in which a SC directly differentiates into a HC without cell division [4, 10–14]. In the mouse cochlear sensory epithelium, HCs are interdigitated by SCs, and the loss of SCs will lead to the death of HCs [15, 16]. Thus, exhausting the supply of SCs to regenerate HCs is not an effective strategy for long-term HC regeneration. In contrast, inducing the SCs to reenter the cell cycle and mitotically generate HCs is a better strategy for HC regeneration. However, in previous reports the majority of regenerated HCs came from direct trans-differentiation, and mitotic HC regeneration only provided a small portion of the newly regenerated HCs.

Our efforts are focused on the promising future of genetic manipulation of two crucial signaling pathways during inner ear development, the Wnt/β-catenin signaling and the Notch signaling pathways [17, 18]. Canonical Wnt/β-catenin signaling is activated by the binding of Wnt ligand to the Frizzled receptor, which stops the ubiquitin-dependent proteolysis of β-catenin that results from the combined action of GSK3β and axin. Accumulated β-catenin then migrates from the cytoplasm to the nucleus where it associates with the TCF/Lef transcription factors and activates its downstream effector genes [19]. Wnt/β-catenin signaling has been reported to be involved in multiple events including proliferation, cell fate determination, differentiation and cell protection [20–24]. Wnt also has a dual function in the developing mouse auditory system by promoting the proliferation of Sox2-positive prosensories and specifying the number of HCs during development [11, 14, 25]. Lgr5 and Axin2 are two Wnt downstream genes in the cochlea [26, 27]. Activation of Wnt/β-catenin signaling initiates the proliferation of Lgr5^+^ cells, which are well established as progenitors for HCs in cochlea [11, 14, 28]. Unfortunately, only a small number of proliferating Lgr5^+^ progenitors trans-differentiate into HCs [10, 14, 29]. This suggests that although Wnt activation can increase the progenitor pool, Wnt activation alone cannot regenerate significant amounts of new HCs and thus is not an ideal approach for HC regeneration.

Notch signaling is well known for its lateral inhibition and feedback mechanisms that influence cell fate during HC differentiation [30]. Previous studies have shown that pharmacological or genetic disruption of Notch receptors such as notch1, Notch ligands such as Dll1 or Jag2, or key factors like γ-secretase lead to the inhibition of Notch signaling and thus induce SC to differentiate into HCs at the cost of exhausting the pool of SCs in both the cochlea and the vestibular organs [31–33]. Mutation or knockdown of downstream transcriptional effectors of Notch signaling, such as members of the *Hes* and *Hey* gene families, also have similar effects [34–39]. However, HCs are interdigitated by the SCs and the loss of SCs will in turn lead to the death of HCs, including the newly differentiated HCs [10, 16, 40]. Thus, Notch inhibition alone is also not an ideal solution for long-term HC regeneration.

Based on previous research on both signaling pathways, we hypothesized that promoting the proliferation of SCs first and then letting the proliferated SCs differentiate into HCs would be the best approach to promote mitotic HC regeneration. Thus, we tested a two-step strategy to activate Wnt/β-catenin signaling first and then inhibit Notch signaling in the mouse cochlea to achieve our goal of mitotically regenerating the HCs. We found that Wnt activation followed by Notch inhibition significantly promotes SCs, including Lgr5^+^ progenitors, to mitotically regenerate new HCs with or without HC damage in the postnatal mouse cochlea.

## RESULTS

### The activation of Wnt/β-catenin signaling induces the proliferation of SCs with or without HC injury

**Figure 1:**
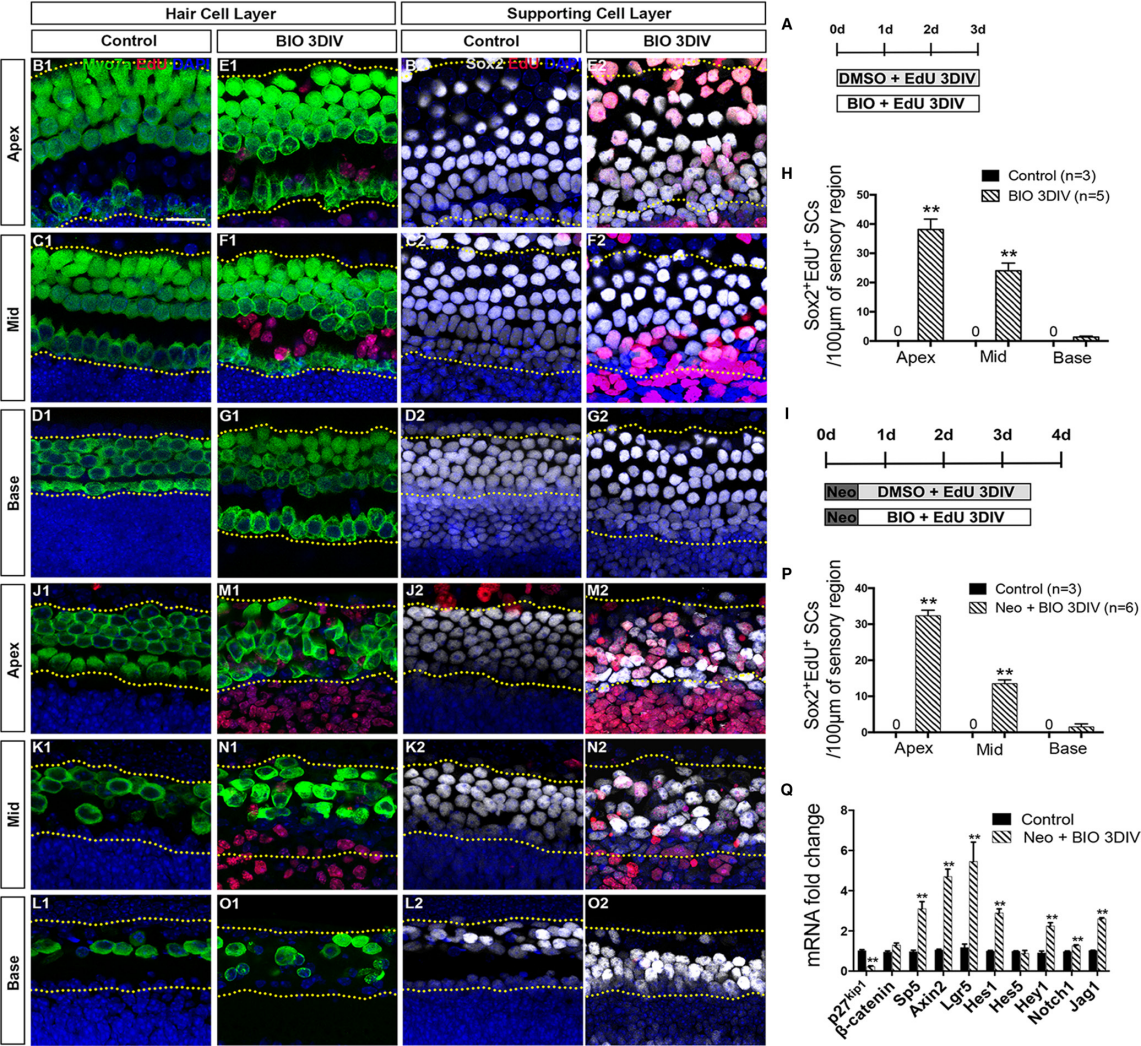
Wnt/β-catenin signaling activation induced proliferation of SCs (**A**) Cochleae were cultured in DMEM/F12 media with 1 mM EdU and 5 μM BIO or 0.5% DMSO for 3 days. (**B**–**G**) Confocal slices from the apex, middle, and base of neonatal organ of Corti explants cultured without neomycin administration. The dotted lines in B–D and E–G showed the counting limits of SCs in the sensory cell region, which includes three rows of Deiters′ cells and inner and outer pillar cells. No Myo7a^+^ (green)/EdU^+^ (red) cells or Sox2^+^ (gray)/EdU^+^ (red) cells were observed in the DMSO-treated cochleae from the apex to the base (Myo7a B1–D1, Sox2 B2-D2). No Myo7a^+^/EdU^+^ cells were observed in the BIO-treated cochleae (E1–G1). Conversely, significant proliferation (Sox2^+^/EdU^+^ cells) was observed in the SC layer in BIO-treated cochleae (E2–G2). DAPI shows the nuclei in blue, and the scale bar is 20 μm. (**H**) The number of Sox2^+^/EdU^+^ cells per 100 μm in the sensory region from the apex to the base of the normal cochleae. (**I**) Cochleae were cultured in DMEM/F12 media with 0.5 mM neomycin overnight at P1, then with 1 mM EdU and 5 μM BIO or 0.5% DMSO for 3 days. (**J**–**O**) Confocal slices from the apex, middle, and base of injured neonatal organ of Corti explants cultured with neomycin. The dotted lines in J–L and M–O showed the counting limits of SCs in the sensory cell region consistent with the uninjured cochleae. No Myo7a^+^ (green)/EdU^+^(red) cells or Sox2^+^ (gray)/EdU^+^ (red) cells were observed in DMSO-treated cochleae from the apex to the base (Myo7a J1–L1, Sox2 J2–L2), while significantly more Sox2^+^/EdU^+^ cells were observed in the BIO-treated group especially in the apex and middle of the cochleae (M2, N2). No Myo7a^+^/EdU^+^ cells were observed in the BIO-treated cochleae (M1–O1). DAPI shows the nuclei in blue, and the scale bar is the same as B1. (**P**) The number of Sox2^+^/EdU^+^ cells per 100 μm in the sensory region from the apex to the base of the injured cochleae. (**Q**) Quantitative RT-PCR results of the mRNA expression changes of Wnt and Notch signaling. Data in G and P are presented as mean ± SEM per 100 μm; **p < 0.01, unpaired Student's *t*-tests (two-tailed), see also Supplementary Table S1.

Previous studies have reported that the activation of Wnt/β-catenin signaling induces the proliferation of Lgr5^+^ progenitors in the neonatal mouse cochlea [11]. Here, we first investigated the detailed effects of Wnt activation in all three turns of cultured mouse cochleae. 6-Bromoindirubin-3′-oxime (BIO), an effective GSK3β inhibitor, was used to pharmacologically activate the Wnt/β-catenin pathway [20, 21, 41–46] *in vitro*. Cochleae were harvested from postnatal day (P)1 C57/BL6 mice and then cultured in DMEM/F12 media with 1 mM EdU and 5 μM BIO or 0.5% DMSO for 3 days (Figure 1A). No Sox2^+^/EdU^+^ cells were observed in the DMSO-treated control group (Figure 1B2–1D2, 1H and Supplementary Table S1). Conversely, significantly more proliferating SCs, indicated by Sox2^+^/EdU^+^ cells, were observed in the apical and middle turns of the BIO-treated cochleae, while very few were seen in the basal turn (Figure 1E2–1G2, 1H and Supplementary Table S1). Moreover, there were no Myo7a^+^/EdU^+^ cells (mitotically regenerated HCs) observed in either the DMSO-treated or BIO-treated cochleae (Figure 1B1–1G1). These data demonstrate that activation of Wnt/β-catenin signaling induces the proliferation of SCs in the apical and middle turns of cultured cochleae but fails to successfully induce the proliferated SCs to differentiate into HCs.

Neomycin is widely used to create the HC damage model [47]. To investigate the detailed effect of Wnt activation in all three turns of the cultured cochleae after HC injury, 0.5 mM neomycin was added into the DMEM/F12 media. This was followed by 1 mM EdU and 5 μM BIO or 0.5% DMSO, and the cochleae were cultured for another 3 days (Figure 1I). No Sox2^+^/EdU^+^ cells were observed in the DMSO-treated group (Figure 1J2–1L2, 1P and Supplementary Table S1), while large numbers of Sox2^+^/EdU^+^ cells were observed in the apical and middle turns of the BIO-treated cochleae (Figure 1M2–1N2, 1P and Supplementary Table S1). However, there was no significant difference in the basal turns between control and BIO-treated cochleae (Figure 1L2, 1O2, 1P and Supplementary Table S1). Consistent with the results in undamaged neonatal cochleae, few Myo7a^+^/EdU^+^ cells were observed in either group after neomycin insult (Figure 1J1–1O1). This result demonstrated that Wnt/β-catenin signaling activation only induced the proliferation of SCs after HC injury and failed to mitotically regenerate significant numbers of new HCs.

Samples from both groups were collected for quantitative RT-PCR to determine the RNA expression levels associated with Wnt and Notch signaling. BIO treatment significantly promoted the expression of the Wnt downstream genes *Sp5, Axin2*, and *Lgr5*, while the cell cycle inhibitor *p27kip1* was inhibited (Figure 1Q). It is interesting that the expression of Notch signaling ligand *Jag1* and downstream genes *Hes1* and *Hey1* were activated as well (Figure 1Q). This suggested that Wnt activation also activated Notch signaling, and the activation of Notch signaling could in turn inhibit the SCs from differentiating into HCs, which might be the reason that Wnt activation alone failed to induce the proliferated SCs to differentiate into HCs. In sum, these data indicate that Wnt/β-catenin signaling activation induces the proliferation of Sox2^+^ SCs in the apical and middle turns of cultured cochleae, and it also activates Notch signaling thus preventing the proliferated SCs from differentiating into HCs.

### Inhibition of Notch signaling after Wnt/β-catenin activation promotes the mitotic regeneration of HCs and increases the total HC number

**Figure 2:**
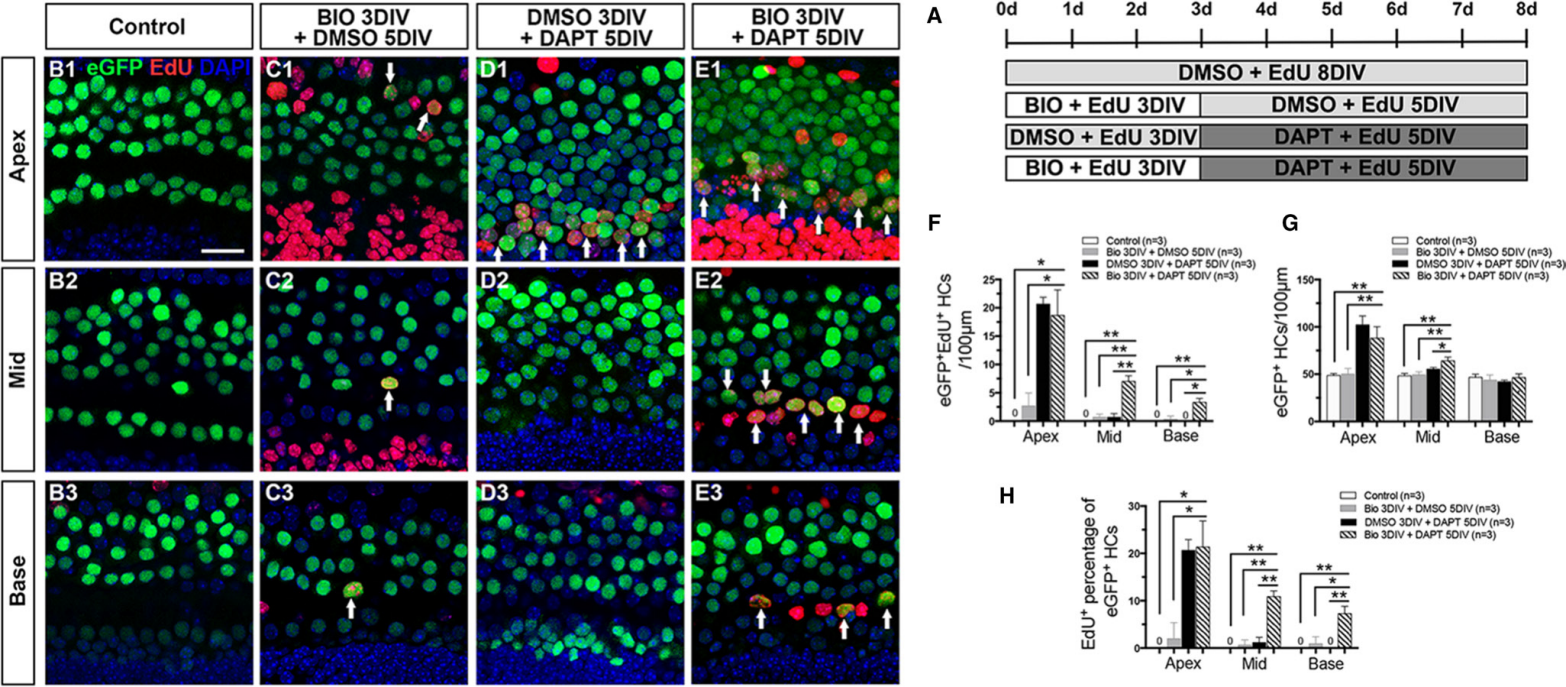
Notch signaling inhibition after Wnt/β-catenin signaling activation increased the number of HCs (**A**) Cochleae of Atoh1-eGFP mice were dissected at P1 for further treatment. In the trial group, 50 μM DAPT (a γ-secretase inhibitor) was added to the culture media for 5 days after BIO treatment. BIO and DAPT were replaced with DMSO in the controls. (**B**–**E**) All Atoh1+ cells were marked by eGFP (green), and EdU (red) was added to the culture media to capture proliferating cells. No eGFP^+^/EdU^+^ cells were observed in the cochleae of the DMSO-treated group (B1–B3). Few eGFP^+^/EdU^+^ cells were observed in the apex of the BIO-DMSO group (C1, arrows). Numerous eGFP^+^/EdU^+^ cells were seen in the apex of the DMSO-DAPT group (D1, arrows). The number of double-positive cells decreased progressively from the apex to the base in the cochleae from the BIO-DAPT group (E1-E3, arrows). DAPI shows the nuclei in blue, and the scale bar is 20 μm. (**F**) The number of eGFP^+^/EdU^+^ cells per 100 μm from the apex to the base of normal cochleae. (**G**) The number of eGFP^+^ cells per 100 μm from the apex to the base of normal cochleae. (**H**) The percentage (%) of EdU^+^ cells of the total eGFP^+^ cells in the normal cochleae. Data in F-H are presented as mean ± SEM per 100 μm; *p < 0.05, **p < 0.01, unpaired Student's *t*-tests (two-tailed), see also Supplementary Table S2.

The experiments above showed that activating Wnt/β-catenin signaling activates Notch signaling and fails to induce the proliferated SCs to differentiate into HCs. Thus we proposed a two-step strategy in which Wnt signaling is first activated to promote SC proliferation and then Notch signaling is inhibited to promote the differentiation of SCs into HCs. In this experiment, we used Atoh1-eGFP transgenic mice in which HCs express eGFP [48]. A γ-secretase inhibitor (DAPT) was added to the culture media after 3 days of BIO treatment (Figure 2A). For analysis, eGFP^+^/EdU^+^ HCs were counted in the HC layer. No eGFP^+^/EdU^+^ cells were observed in the DMSO-treated control cochleae (Figure 2B1–2B3, 2F and Supplementary Table S2). Very few eGFP^+^/EdU^+^ cells, which represent the mitotically regenerated HCs, were observed in the BIO-DMSO group (Figure 2C1–2C3, 2F and Supplementary Table S2), but significant numbers of eGFP^+^/EdU^+^ cells were observed in the apex of the DMSO-DAPT group (Figure 2D1, 2F and Supplementary Table S2). In contrast, the BIO-DAPT-treated cochleae had significantly more eGFP^+^/EdU^+^ cells in all three turns compared with the BIO-DMSO and control groups (Figure 2E1–2E3, 2F and Supplementary Table S2) and in the middle and basal turns compared to the DMSO-DAPT group (Figure 2F and Supplementary Table S2). The number of eGFP^+^/EdU^+^ cells decreased from the apex to the base (Figure 2F). In addition, BIO-DAPT-treated cochleae had significantly more eGFP^+^ HCs in the apical and middle turns compared to the BIO-DMSO and control groups (Figure 2G and Supplementary Table S2). When we compared the ratio of mitotically regenerated HCs in all eGFP^+^ HCs, we found that BIO-DAPT-treated cochleae had significantly higher percentages of eGFP^+^/EdU^+^ HCs in all three turns compared with the BIO-DMSO and control groups and in the middle and basal turns compared with the DMSO-DAPT group (Figure 2H and Supplementary Table S2). To further verify this finding, we used another HC maker, Pou4f3, in a separate experiment. Pou4f3 staining also showed the similar result that there are significantly more Pou4f3^+^/EdU^+^ HCs in the middle and basal turns of BIO-DAPT-treated cochleae compared with the DMSO-DAPT group without neomycin (Supplementary Figure S2 and Supplementary Table S8). These results demonstrated that suppressing Notch signaling following Wnt activation could significantly promote the mitotic regeneration of HCs and increase the total HC number.

**Figure 3:**
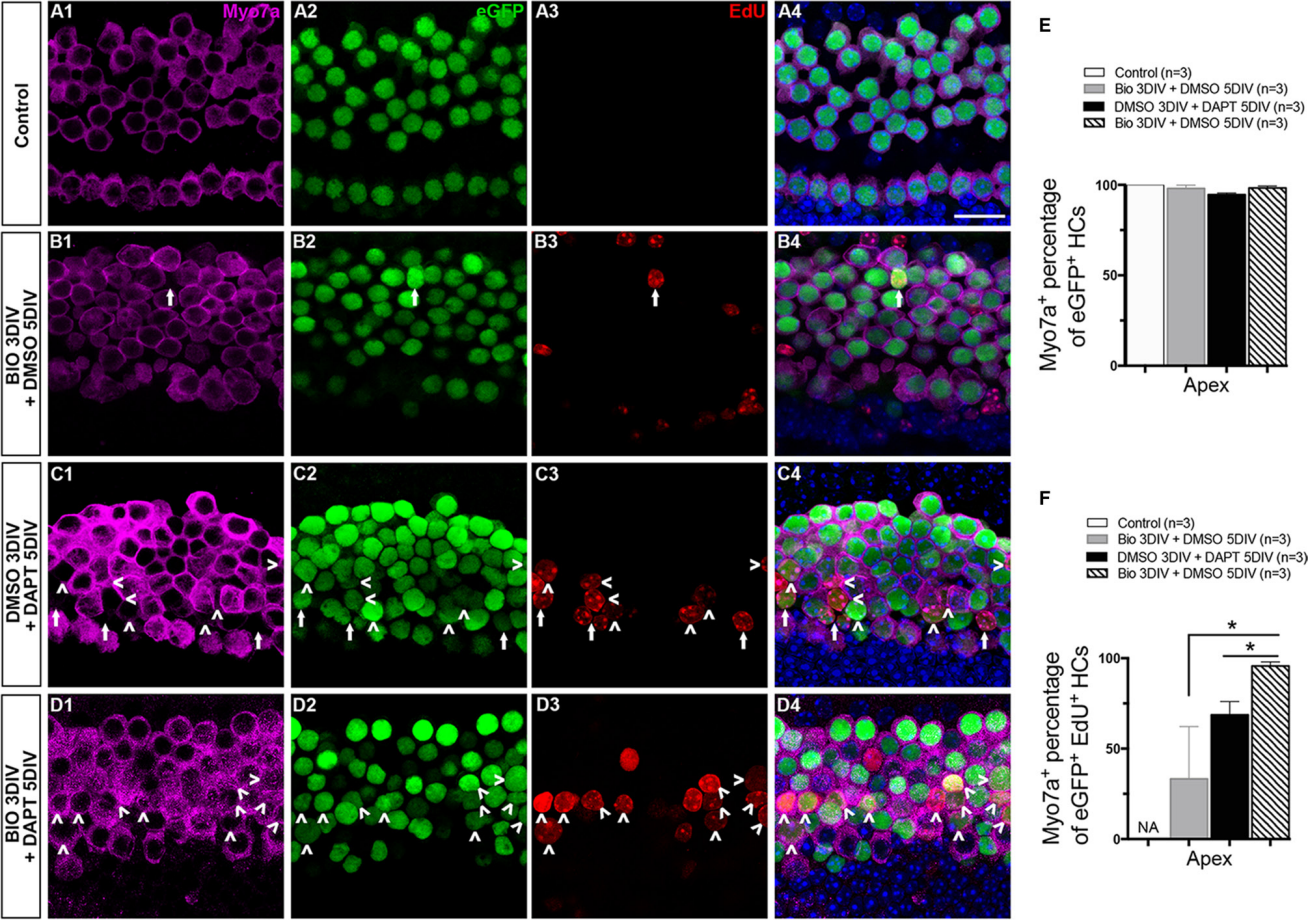
The maturation of mitotically regenerated HCs (**A**–**D**) To determine whether the newly generated eGFP^+^ cells were mature HCs, cochleae were stained with antibodies against Myo7a. All images are from the apex of the injured cochleae. In the control group, all HCs were co-marked by both eGFP^+^ (green) and Myo7a^+^ (magenta) (A4). The situation was nearly the same in the BIO-DMSO group except for the occasional appearance of an eGFP^+^/EdU^+^ cell (green and red, B4). Numerous eGFP^+^ cells without Myo7a expression were seen in the DMSO-DAPT group, and many of these were eGFP^+^/EdU^+^ cells (C1–C4). Most of the eGFP^+^ cells and eGFP^+^/EdU^+^ cells in the BIO-DAPT group were co-marked by Myo7a (D1–D4). Arrows show Myo7a-/eGFP^+^/EdU^+^ cells and arrowheads show Myo7a^+^/eGFP^+^/EdU^+^ cells. DAPI shows the nuclei in blue, and the scale bar is 20 μm. (**E**–**F**) Graphs E and F show the percentages of Myo7a^+^ cells in eGFP^+^ cells and EdU^+^/eGFP^+^ cells, respectively, in the apex of the cochleae. Data are represented as mean ± SEM; *p < 0.05, unpaired Student's *t*-tests (two-tailed), see also Supplementary Table S3.

The experiments above showed that both the BIO-DAPT group and the DMSO-DAPT group had significant numbers of eGFP^+^/EdU^+^ cells, and majority of the eGFP^+^/EdU^+^ cells were in the apex (Figure 2D1, 2E1, 2F, and Supplementary Table S2). To determine whether these mitotically regenerated eGFP^+^ HCs in the apex were mature HCs, the cochleae were stained with antibodies against Myo7a and Prestin. Myo7a is a more mature HC marker than Atoh1, and Prestin is a mature outer HC marker that begins to be expressed at P6. In the control group, all eGFP^+^ cells in the apex were co-labeled with Myo7a, and no eGFP^+^/EdU^+^ cells were observed (Figure 3A1–3A4, 3E, and 3F). In the BIO-DMSO group, the majority of eGFP^+^ cells were co-labeled with Myo7a in the apex (Figure 3B1–3B4, and 3E). Only a few eGFP^+^/EdU^+^ cells were observed, and around 33.33 ± 16.67% of these were also Myo7a^+^ (Figure 3B1–3B4, 3F, and Supplementary Table S3). In the DMSO-DAPT group, some eGFP^+^ cells were without Myo7a labeling (Figure 3C1–3C4 and 3E). A significant number of eGFP^+^/EdU^+^ cells were observed, but only around 68.72 ± 7.29% of them were co-labeled with Myo7a (Figure 3C1–3C4, 3F, and Supplementary Table S3). In contrast, it was very interesting that most of the eGFP^+^ cells and 95.67 ± 2.27% of the eGFP^+^/EdU^+^ cells in the BIO-DAPT group were also Myo7a^+^ (Figure 3D1–3D4, 3E, 3F, and Supplementary Table S3). These data suggested that Notch signaling inhibition after Wnt/β-catenin signaling activation might help to promote the maturation of new mitotically regenerated HCs compared to Notch inhibition alone. To further verify this finding, in a separate experiment, we extended the culture duration from 8 days to 10 days, and stained with the mature HC maker Prestin. We found that after 10 days culture, Prestin^+^/EdU^+^ HCs were observed in both DMSO-DAPT group and BIO-DAPT group; however, the number of Prestin^+^/EdU^+^ HCs had no significantly difference between the two groups (Supplementary Figure S3C2–S3D2, S3E and Supplementary Table S9).

### After neomycin-induced injury, Wnt/β-catenin activation followed by Notch inhibition promotes the mitotic regeneration of HCs and increases the HC number in neonatal cochleae

**Figure 4:**
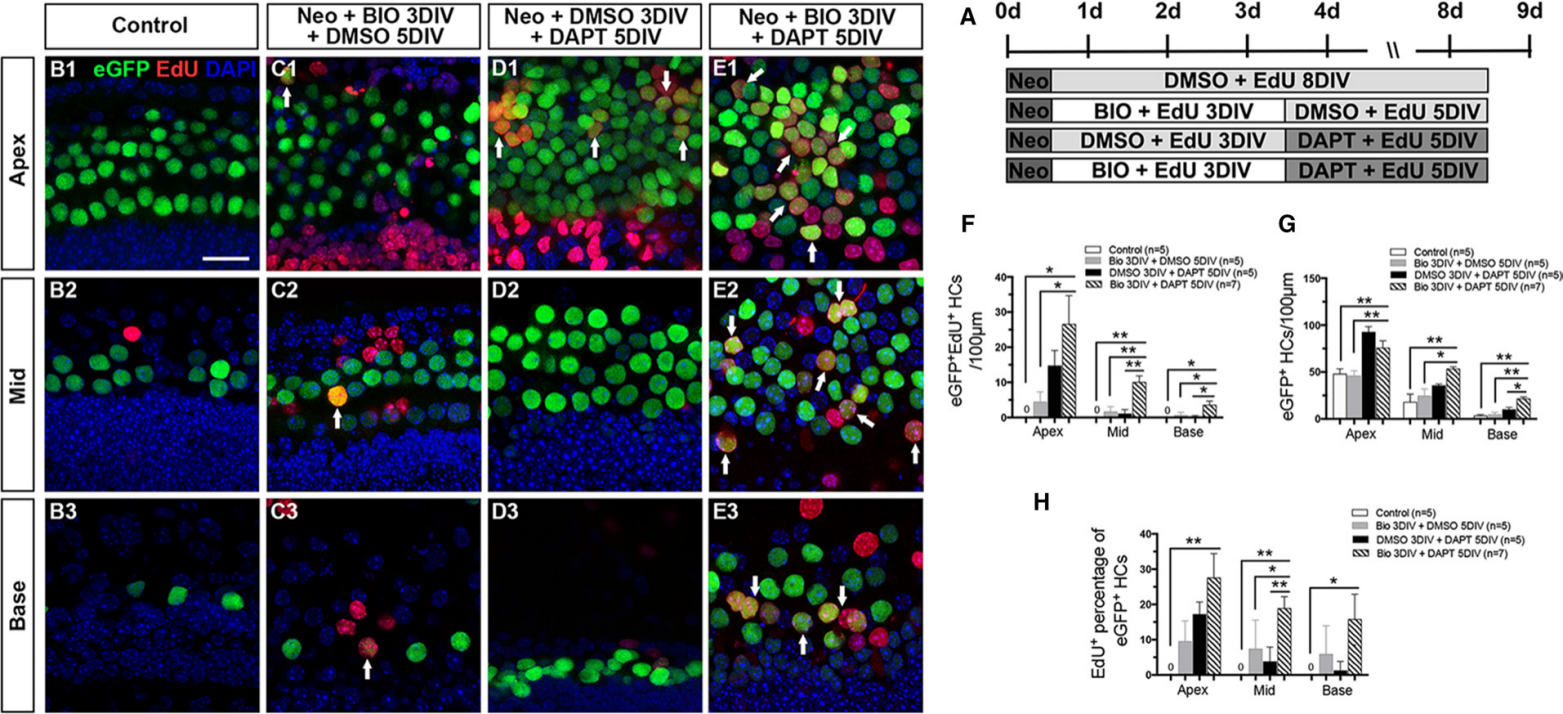
Wnt/β-catenin signaling activation followed by Notch signaling inhibition increased HC regeneration in neonatal mouse cochleae after damage (**A**) Cochleae of Atoh1-eGFP^+^ mice were cultured in DMEM/F12 media with 0.5 mM neomycin overnight at P1, then with 1 mM EdU and 5 μM BIO or 0.5% DMSO for 3 days followed by 50 μM DAPT or 0.5% DMSO for 5 more days. (**B**–**E**) Consistent with the results in the normal neonatal cochleae, no eGFP^+^/EdU^+^ cells were observed in the DMSO-treated group (green and red, B1–B3), and very few eGFP^+^/EdU^+^ cells were observed in the BIO-DMSO group (C1–C3, arrows). Several eGFP^+^/EdU^+^ cells were observed in the apex of the DMSO-DAPT group (D1, arrows). Greater numbers of eGFP^+^/EdU^+^ cells were observed in the BIO-DAPT group (E1–E3, arrows). DAPI shows the nuclei in blue, and the scale bar is 20 μm. (**F**) The number of eGFP^+^/EdU^+^ cells per 100 μm from the apex to the base of the injured cochleae. (**G**) The number of eGFP^+^ cells per 100 μm from the apex to the base of the injured cochleae. (**H**) The percentage (%) of EdU^+^ cells of the total eGFP^+^ cells in the injured cochleae. Data in F–H are represented as mean ± SEM per 100 μm; *p < 0.05, **p < 0.01, unpaired Student's *t*-tests (two-tailed), see also Supplementary Table S4.

To investigate whether the two-step strategy could also promote the mitotic regeneration of HCs and increase the total HC number after HC injury *in vitro*, cultured cochleae were injured with neomycin at P0 for 12 hours (Figure 4A). No eGFP^+^/EdU^+^ cells were observed in the DMSO control group (Figure 4B1–4B3, 4F and Supplementary Table S4). A few eGFP^+^/EdU^+^ cells were observed in the BIO-DMSO and the DMSO-DAPT-treated cochleae, and these appeared mainly in the apex (Figure 4C1–4C3, 4D1–4D3, 4F and Supplementary Table S4). Interestingly, significantly more eGFP^+^/EdU^+^ cells were observed in the BIO-DAPT group in all three turns compared with the control and BIO-DMSO groups, and the BIO-DAPT-treated group also had significantly more eGFP^+^/EdU^+^ cells than the DMSO-DAPT group in the middle and basal turns (Figure 4E1–4E3, 4F and Supplementary Table S4). The number of eGFP^+^/EdU^+^ cells also decreased from the apex to the base (Figure 4F). Moreover, after HC injury, BIO-DAPT-treated cochleae had significantly more total eGFP^+^ HCs in all three turns compared to the BIO-DMSO and control groups and in the basal turn compared to the DMSO-DATP group (Figure 4G and Supplementary Table S4). When we compared the ratio of mitotically regenerated HCs, we found that BIO-DAPT-treated cochleae also had the highest percentages of eGFP^+^/EdU^+^ HCs among all eGFP^+^ HCs in all three turns compared with all the other groups after HC injury (Figure 4H and Supplementary Table S4). These results demonstrated that after neomycin-induced HC loss Wnt activation followed by Notch inhibition could significantly promote the mitotic regeneration of HCs and increase the total HC number.

### The SC number in the BIO-DAPT group is partially restored compared with the DMSO-DAPT group

**Figure 5:**
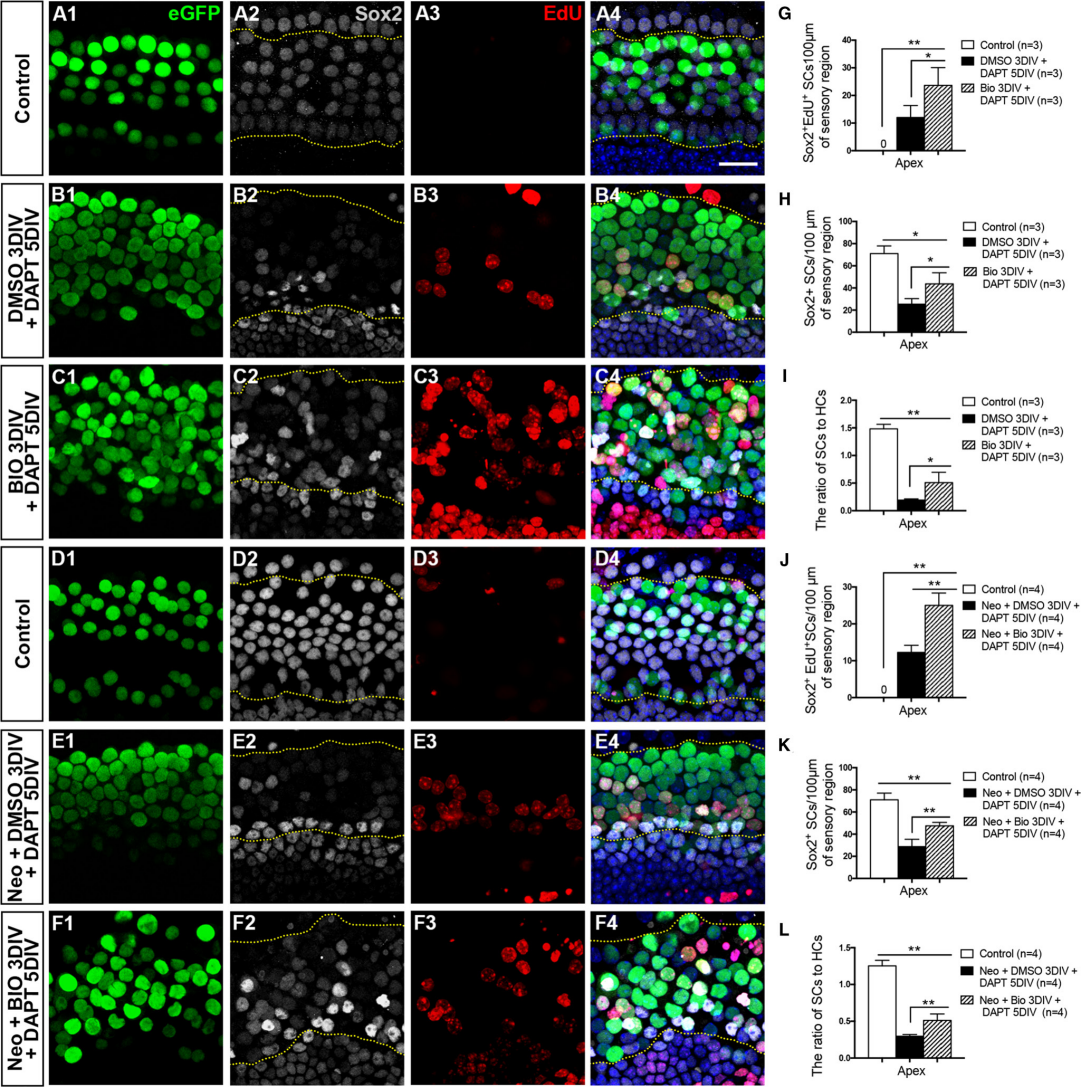
SC proliferation was promoted and the SC number was partially restored in the BIO-DAPT group (**A**–**C**) SCs were counted and analyzed. Images were taken from the apex of the uninjured cochleae. The dotted lines in A2–C2 and A4–C4 showed the counting limits of SCs in the sensory cell region. HCs (eGFP^+^, green) and SCs (Sox2^+^, gray) were well organized and integrated in the control group (A1–4). Sox2 expression was significantly decreased in the SC region in the DMSO-DAPT group (B1–B4). In contrast, Sox2 expression was preserved to a certain degree in the BIO-DAPT group (C1–C4). EdU is red, DAPI is blue, and the scale bar is 20 μm. (**D**–**F**) Images were taken from the apex of the cochleae after neomycin administration. The dotted lines in D2–F2 and D4–F4 showed the counting limits of SCs in the sensory cell region consistent with the uninjured cochleae. The numbers of HCs (eGFP^+^, green) and SCs (Sox2^+^, gray) were similar to normal cochleae in the control group (D1–D4). Sox2 expression was weaker in the SC region in the DMSO-DAPT group (E1–E4), while it was preserved to a certain degree in the BIO-DAPT group (F1–F4). EdU is red, DAPI is blue, and the scale bar shares the same in A4. (**G**–**J**) Graphs G and J show the number of Sox2^+^/EdU^+^ cells per 100 μm in the sensory region of the injured (J) or uninjured (G) cochleae. Graphs H and K show the number of Sox2^+^ cells per 100 μm in the sensory region of the injured (**K**) or uninjured (H) cochleae. Graphs I and L show the ratio of Sox2^+^ SCs to eGFP^+^ HCs in the apex of the injured (**L**) or uninjured (I) cochleae. Data are presented as mean ± SEM; *p < 0.05, **p < 0.01, unpaired Student's *t*-tests (two-tailed), see also Supplementary Table S5.

Previous studies have shown that inhibition of Notch signaling can induce the differentiation of SCs and that this leads to an increased number of HCs with a concomitant loss of SCs [31, 49, 50]. However, without considerable proliferation of SCs, the loss of SCs will lead to the eventual death of the regenerated HCs [15, 16]. Our experiments described above showed that with or without HC injury the majority of the mitotically regenerated HCs were in the apex, and both the BIO-DAPT group and DMSO-DAPT group had significant amounts of newly regenerated HCs in the apex with no significant difference between the two groups (Figure 2F and 2G, Figure 4F and 4G, Supplementary Tables S2 and S4). Thus, we next investigated the proliferation of SCs in the apical turns of the cochlea in both the BIO-DAPT and DMSO-DAPT groups. We counted the Sox2^+^ SCs in the organ of Corti (between the dashed lines in Figure 5 A2-F2 and A4-F4) and observed a few Sox2^+^/EdU^+^ SCs (Figure 5A4–5F4), and the number of SCs decreased dramatically in the apex of the DMSO-DAPT group both with and without HC injury (Figure 5B1–4, 5E1–4, 5G–5H, 5J–5K and Supplementary Table S5). In contrast, in the apex of the BIO-DAPT group we found significantly more Sox2^+^/EdU^+^ SCs, and the total SC number also increased to some extent compared to the DMSO-DAPT group with or without HC injury (Figure 5C1–4, 5F1–4, 5G–5H, 5J–5K and Supplementary Table S5). We found that the BIO-DAPT group also had a significantly higher SC/HC ratio than the DMSO-DAPT group in the apex with or without HC injury (Figure 5I, 5L and Supplementary Table S5), suggesting that the HCs in the BIO-DAPT group might be better supported by the SCs. These results indicate that our two-step strategy could regenerate HCs while partially preserving the SC number, which might aid the survival of HCs. Thus, the two-step strategy might be a better approach for long-term HC regeneration.

### The majority of proliferative SCs in the BIO group originate from Lgr5^+^ progenitor cells

**Figure 6:**
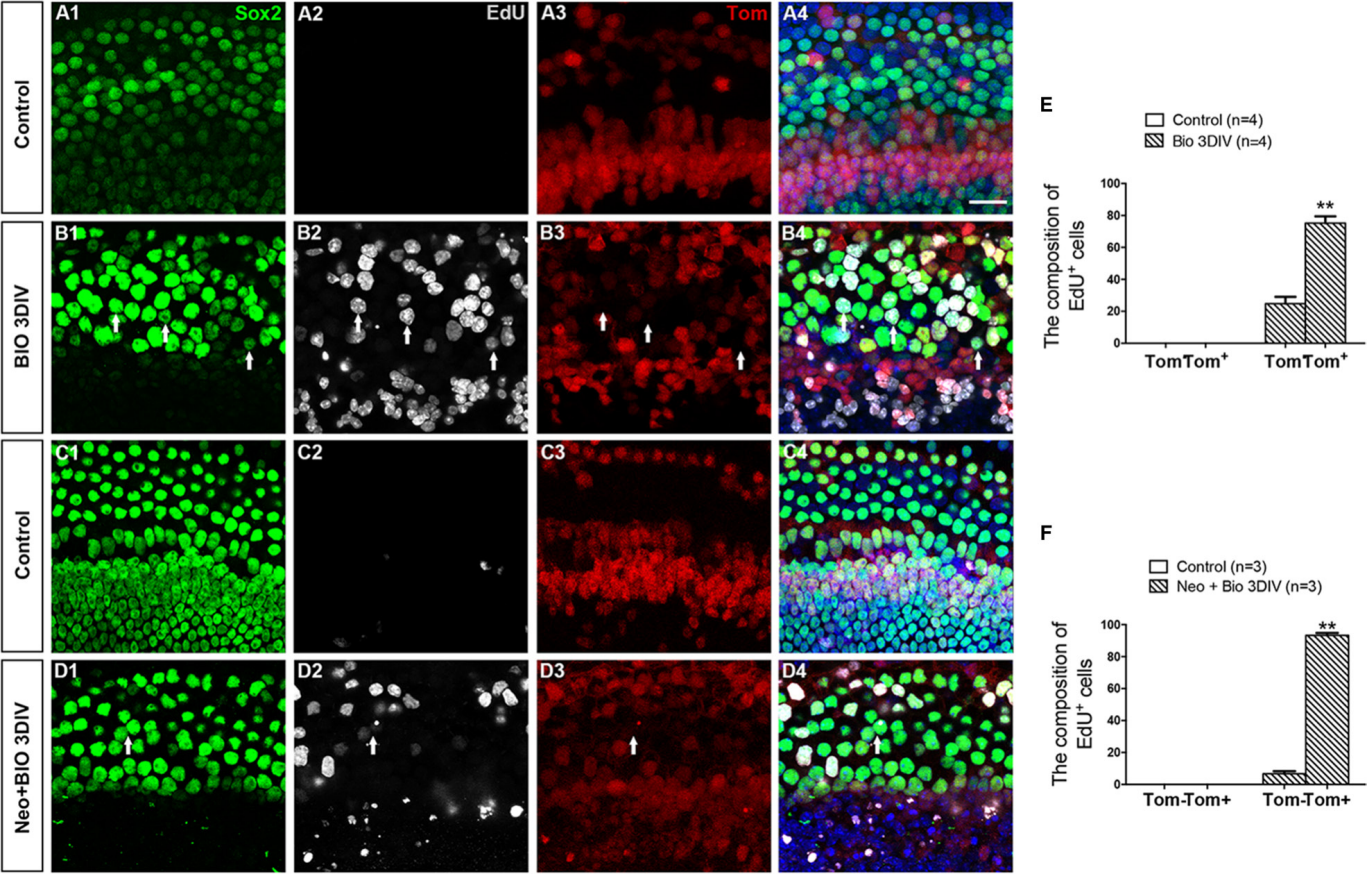
The majority of proliferative SCs in the BIO-treated group originated from Lgr5+ progenitor cells (**A**–**D**) Rosa26 reporter mice were crossed with Lgr5^eGFP-CreER^ mice for lineage tracing. All images are from the apex of the normal (A, B) and injured (C, D) cochleae. SCs were marked by Sox2 (green, A1–D1), and proliferative cells were marked by EdU (Gray, A2–D2). All Lgr5^+^ cells expressed tdTomato (Tom, red, A3-D3). Few Sox2^+^/Tom^+^ cells were seen in control groups (A4, C4). A total of 75.22 ± 4.23% of the proliferative Sox2^+^ SCs in the apex had originated from Lgr5^+^ SCs in normal cochleae (B1–B4), and 93.26 ± 1.57% of the Lgr5^+^ SCs in the neomycin-injured cochleae expressed tdTomato after BIO administration (D1–D4). Arrows show Tom^−^/Sox2^+^/EdU^+^ cells. DAPI is blue and the scale bars are 20 μm. (**E**–**F**) The graphs show the ratios of Tom- and Tom+ cells in Sox2^+^/EdU^+^ cells of the normal (E) and neomycin-treated (F) cochleae. Data are presented as mean ± SEM; **p < 0.01, unpaired Student's *t*-tests (two-tailed), see also Supplementary Table S6.

To identify the source of the proliferating SCs in the BIO group, we crossed the Lgr5^eGFP-CreER^ mice to loxp floxed reporter Rosa26-tdTomato mice to lineage trace the Lgr5^+^ progenitor cells. Because the apex had the most proliferated SCs, we chose the apex as the representative material for this experiment. We found no Sox2^+^/EdU^+^ cells in the control group with or without HC injury (Figure 6A1–6A4, 6C1–6C4, and Supplementary Table S6), but we found significant numbers of Sox2^+^/EdU^+^ cells in the BIO group both with and without damage (Figure 6B1–6B4, 6D1–6D4, and Supplementary Table S6). Without HC injury, 75.22 ± 4.23% of the Sox2^+^/EDU^+^ cells were tdTomato^+^, and with HC injury 93.26 ± 1.57% of them were tdTomato^+^ (Figure 6E–6F, and Supplementary Table S6). This suggested that the majority of the proliferative SCs generated by Wnt activation had originated from Lgr5^+^ progenitor cells.

### The majority of the mitotically regenerated HCs in the BIO-DAPT group originate from Lgr5^+^ progenitor cells

**Figure 7:**
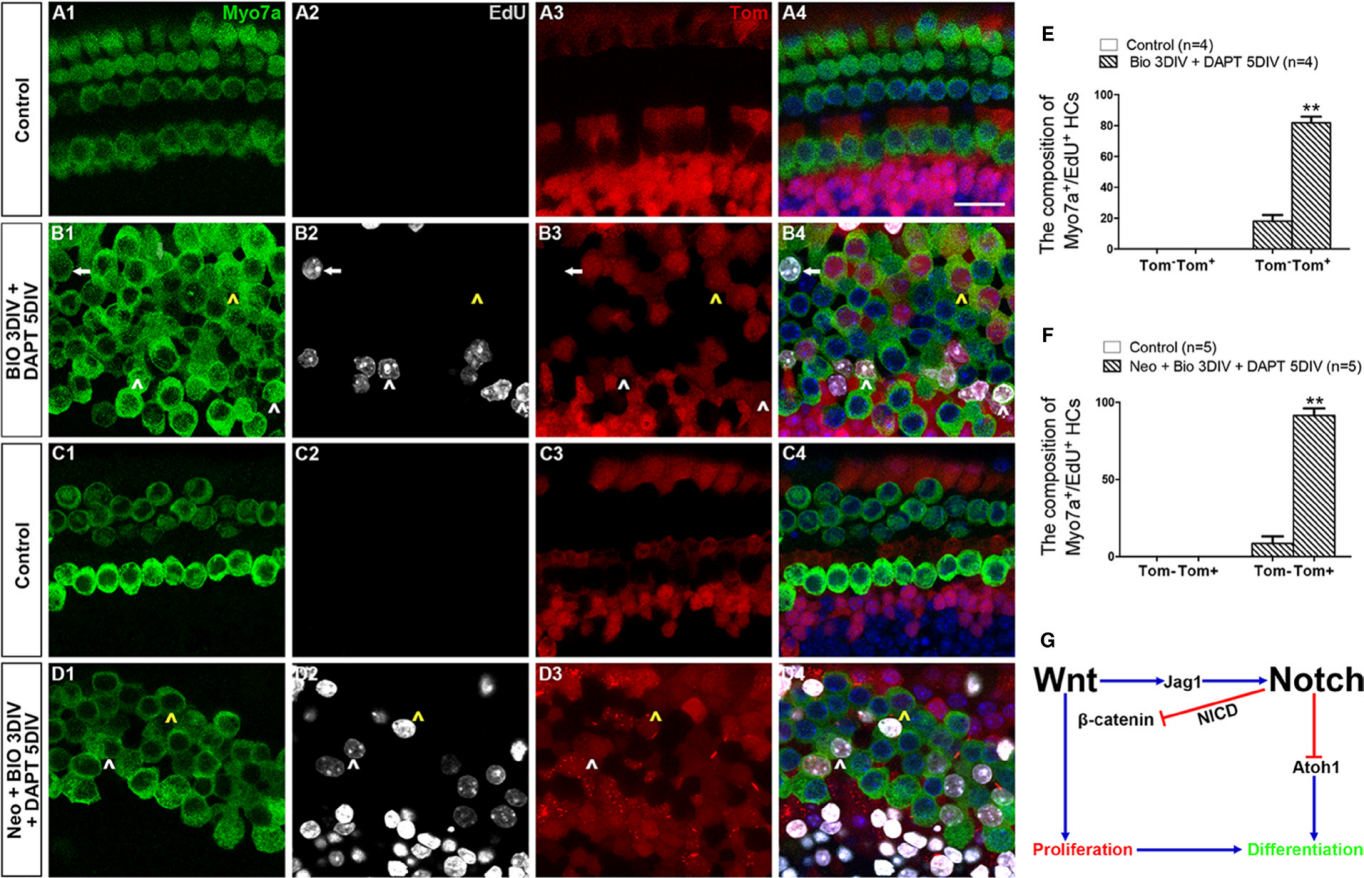
The majority of mitotically regenerated HCs in the BIO-DAPT group originated from Lgr5^+^ progenitor cells (**A**–**D**) Rosa26 reporter mice were crossed with Lgr5^eGFP-CreER^ mice for lineage tracing. All images are from the apex of the normal (A, B) and injured (C, D) cochleae. HCs were marked by Myo7a (green, A1–D1), and proliferating cells were marked by EdU (Gray, A2–D2). All Lgr5^+^ cells expressed tdTomato (Tom, red, A3–D3). All images are from the apex of the cochleae. Few Myo7a^+^/Tom^+^ cells were seen in the control groups (A4, C4). A large proportion of the Myo7a^+^/EdU^+^ cells (81.90 ± 3.90%) in the normal BIO-DAPT-treated cochleae expressed tdTomato (B4), and 91.47 ± 4.66% of the Myo7a^+^/EdU^+^ cells were marked by tdTomato in the injured cochleae (D4). White arrowheads show Tom^+^/Myo7a^+^/EdU^+^ cells, and arrows show Tom^−^/Myo7a^+^/EdU^+^ cells. Moreover, there were several EdU^−^/Myo7a^+^/Tom^+^ cells in both the normal and injured cochleae that differentiated from the original SCs (B4, D4, yellow arrowheads). DAPI is blue and the scale bars are 20 μm. (**E**–**F**) The graphs show the ratio of Tom^−^ and Tom^+^ cells in Myo7a^+^/EdU^+^ cells of normal (E) and neomycin-treated (F) cochleae. Data are presented as mean ± SEM; **p < 0.01, unpaired Student's *t*-tests (two-tailed), see also Supplementary Table S6. (**G**) Schematic of the possible crosstalk between Wnt and Notch signaling during the proliferation and differentiation process.

To identify the origin of the new mitotically regenerated HCs, we used the Lgr5-eGFP-CreER/Rosa26-tdTomato mice for lineage tracing. We also used the apex as the material in this experiment because most of the mitotically regenerated HCs were in the apex. We found no Myo7a^+^/EdU^+^ cells in the control group with or without damage (Figure 7A1–7A4, 7C1–7C4, Supplementary Table S6), but we found a significant number of Myo7a^+^/EdU^+^ cells in the BIO-DAPT group both with and without damage (Figure 7B1–7B4, 7D1–7B4, Supplementary Table S6). Without HC injury, 81.90 ± 3.90% of the Myo7a^+^/EdU^+^ cells were tdTomato^+^, and with HC injury 91.47 ± 4.66% of them were tdTomato^+^ (Figure 7E–7F, Supplementary Table S6), and this suggested that these mitotically regenerated HCs had originated from Lgr5^+^ progenitor cells. Together, these results demonstrate that Wnt activation followed by Notch inhibition induces proliferation within a subset of SCs (the Lgr5^+^ progenitor cells) and that these proliferated Lgr5^+^ progenitor cells then undergo subsequent differentiation into HCs.

## DISCUSSION

Multiple studies have shown that activation of the Wnt/β-catenin signaling pathway can induce the proliferation of Lgr5^+^ progenitors in neonatal cochleae, and a small number of these proliferative Lgr5^+^ progenitors can subsequently differentiate into HCs *in vitro* [11, 51, 52]. Our results also confirm that activation of the Wnt/β-catenin signaling pathway can induce the proliferation of SCs with or without HC injury. Also consistent with previous reports, our lineage tracing results showed that most of the proliferative SCs originated from Lgr5^+^ progenitor cells [11, 52, 53]. In this study we used BIO as a Wnt agonist to activate the Wnt signaling. BIO has been reported as an effective inhibitor for GSK3β, which activates canonical Wnt signaling and promotes cell proliferation in plenty of studies on different cell lines and auditory systems [20, 21, 41–46, 54]. Although, as an inhibitor for GSK3 α/β, BIO also inhibits family of cyclin dependent kinases (CDKs), especially CDK5, in neurons [55]. Application of CDK5 inhibitors or expression of CDK inhibitors (CKIs) like p27Kip1, can trigger the differentiation of precursor cells in auditory development [56, 57]. And inhibition of the CKIs of the Cip/Kip family (p21Cip1, p27Kip1, and p57Kip2), especially the inhibition of p27Kip1, allow SC proliferation to occur [58]. However, there are no similar reports about BIO as an inhibitor for GSK3 α/β also inhibits CDKs in auditory organs.

There is evidence that BIO as an effective inhibitor for GSK3β activates wnt signaling and promotes cell proliferation via activating *Jag1*/Notch signaling and inhibiting *p27Kip1* in cochlea [59, 60]; here, we also observed the similar results. Our qRT-PCR results with the injured cochleae also showed that after Wnt activation with BIO treatment, the expression of *Jag1*, *Hes1*, and *Hey1* in the Notch signaling pathway were also activated. Notch signaling has been well studied in inner ear development and prevents the surrounding SCs from differentiating into HCs via lateral inhibition [50, 61–64]. Thus the activation of Notch signaling upon Wnt activation likely prevents the newly proliferated SCs from differentiating into HCs. Here, we found that very few eGFP^+^/EdU^+^ cells were observed in the cochleae of the BIO-DMSO group with or without HC injury which also supports the notion that most of the proliferated SCs fail to differentiate into HCs after Wnt signaling activation alone. The Wnt correlated Notch signaling activation might also promote SCs proliferation and be responsible for preventing the proliferative SCs from differentiating (Figure 7G) [4, 60, 65, 66]. Coincident with the activation of Notch-related mRNA, our qRT-PCR results showed that BIO induced Wnt activation also accompanied with inhibition of *p27Kip1*, which would promote the proliferation of SCs [59, 67]. Taken together, activation of Wnt/β-catenin signaling can promote SCs proliferation but it alone was not able to initiate the differentiation of SCs.

Moreover, previous studies reported that after Wnt activation, the number of proliferated Lgr5^+^ progenitors decreased at P21, suggesting that most of the proliferated Lgr5^+^ progenitors eventually undergo cell death without further differentiation *in vivo* [11]. This is supported by our observation that there was a significant decrease in the number of proliferated SCs from 3 days to 8 days after Wnt activation (Supplementary Figure S1, Supplementary Table S7). This process might be related to the activation of the cell-death pathway caused by the absence of retinoblastoma [40] or the activation of JNK signaling stimulated by non-canonical Wnt signaling via Dishevelled (Dvl) [68, 69]. Therefore, Wnt/β-catenin activation alone does not lead to successful HC regeneration in postnatal mice.

It is well established that the inhibition of Notch signaling is critical for the proper development of HCs and can induce the regeneration of HCs at the cost of exhausting the pool of SCs [32, 50, 64]. However, HCs, including the newly regenerated HCs, will also undergo cell death in absence of SCs [10, 16]. In this study, the number of HCs in the cochleae from the DMSO-DAPT group was also significantly increased accompanied by a sharp decrease in SC number. Although several mitotically regenerated HCs were also observed, which was consistent with our own recent report that inhibition of Notch signaling in Sox2^+^ SCs initiates mitotic regeneration of HCs [4]. Thus, Notch inhibition alone is also not an ideal strategy for long-term HC regeneration.

The failure of either of the two strategies above to induce HC regeneration on their own is the most important reason that current HC regeneration strategies have moved away from focusing on manipulating individual pathways or genes to the co-regulation of multiple pathways. A recent report in zebrafish also showed that localized interactions between the Notch and Wnt Pathways are required during regeneration of sensory hair cells [70]. In a separate experiment, we tried to stimulate Wnt signaling and suppress Notch signaling on neonatal transgene mice simultaneously. However, we found that simultaneously co-regulation of Wnt and Notch signaling produces tremendous proliferation within the prosensory domain without sufficient corresponding mitotic HC regeneration. These results led us to our two-step strategy in which we have used sequential manipulation of these two vital pathways, activating Wnt signaling first then inhibiting Notch signaling, to achieve a significant increase of the mitotic regeneration of HCs. We hypothesized that the ideal strategy for HC regeneration is to regenerate the HCs while preserving the SC number and this would require promoting SC proliferation first and then inducing the proliferated SCs to differentiate into HCs. Thus, we tested our strategy in which activated Wnt/β-catenin signaling promotes SC proliferation first and then inhibition of Notch signaling induces the differentiation of proliferated SCs into HCs. Despite the fact that EdU is unable to capture all of the proliferative cells due to its very short half-life, it is still very exciting that a considerable number of mitotically regenerated HCs were observed in the cochleae of the BIO-DAPT group with or without neomycin-induced HC injury. The total HC numbers in both the DMSO-DAPT and BIO-DAPT groups were significantly greater than the DMSO and BIO-DMSO groups with or without HC injury. Compared to the DMSO-DAPT group, the numbers of mitotically regenerated HCs and the ratio of mitotically regenerated HCs to all HCs were significantly higher in BIO-DAPT group. These results implied that combined with Wnt/β-catenin signaling activation, the occurrence of mitotic HC regeneration that primarily occurred in the apex in the DMSO-DAPT group could expand to the middle and basal turns. Our two-step strategy turns out to be more efficient than either individual treatment to promote mitotic HC regeneration.

However, similar to previous reports [10, 71], the newly regenerated HCs described in our two-step strategy still underwent incomplete maturation. Although almost all Atoh1-eGFP^+^/EdU^+^ cells could be marked by the HC marker Myo7a, none of them could be labeled with the mature outer HC marker Prestin (data not shown). One possible reason for this could be that Prestin is a mature outer HC marker that is only starting to be expressed in outer HCs at P6. We cultured the P1 cochlea for 3 days with Wnt/β-catenin activation to induce SC proliferation and then for 5 days with Notch inhibition to regenerate HCs. Thus, even though the new HCs began to regenerate on the first day of Notch inhibition, 5 days might still not be long enough for the new HCs to be mature enough to express Prestin. When we longer the DAPT administration span from 5 days to 7 days, about 30% of Pou4f3^+^/EdU^+^ cells could be marked by Prestin. In addition, due to the conditions used to culture the whole cochleae, we could not observe the cochleae for longer time or with other factors that might be necessary for HCs to become fully mature. It would be of great interest to explore whether our two-step strategy would lead to better outcomes in *in vivo* experiments in the future. Transduction of Espn gene may also provide a new sight to promote regenerated HCs maturation [72].

To determine the origin of the mitotically regenerated HCs, we lineage traced the Lgr5^+^ progenitors, and found the majority of mitotically regenerated HCs originated from the Lgr5^+^ progenitors in both injured and uninjured cochleae. This result further demonstrated that Lgr5^+^ progenitors are the most promising candidates for HC regeneration.

In summary, activation of Wnt/β-catenin signaling can induce the proliferation of SCs, including Lgr5^+^ progenitor cells. After this proliferation, Notch signaling inhibition can induce the proliferated SCs/Lgr5^+^ progenitor cells to mitotically regenerate new HCs. Our results support our hypothesis that Wnt/β-catenin signaling activation followed by Notch signaling inhibition promotes mitotic regeneration of new HCs in the cochlea while partially preserving the SC number with or without HC damage. This study provides a promising two-step approach for the mitotic regeneration of HCs *in vitro*, and this approach would be a good basis for studying *in vivo* HC regeneration in mammalian animal experiments in the future.

## MATERIALS AND METHODS

### Animals

We used C57BL/6J wild type mice and three types of transgenic mice. Atoh1-EGFP [48] mice were obtained from Jane Johnson (University of Texas Southwestern Medical Center, Dallas), and Lgr5^eGFP-CreER^ (Stock 008875) [73] and Rosa26-tdTomato (Stock 007914) [74] mice were purchased from The Jackson Laboratory. The Atoh1-eGFP, Lgr5^eGFP-CreER^, and Rosa26-tdTomato mice were genotyped with PCR (Table S10). All animal experiments were approved by the Institutional Animal Care and Use Committee of Fudan University.

### Cochlear explant cultures

Cochleae from postnatal day one (P1) mice were dissected in phosphate buffered saline (PBS, Hyclone) and cultured in DMEM/F12 (Hyclone) with 1% N2 supplement (Life Technologies), 2% B27 supplement (Life Technologies), and 50 μg/ml ampicillin (Sigma) at 37°C with 5% CO_2_. The explanted cochlea were treated with 0.5 mM neomycin sulfate (Sigma), 5 μM BIO-Acetoxime (Sigma), or 50 μM DAPT (γ-Secretase inhibitor IX). A final concentration of 10 μM EdU (5′-ethynyl-2′-deoxyuridine, Life Technologies) was added to the culture media throughout the entire culture period to label proliferative cells. Because BIO and DAPT were dissolved in DMSO (Sigma), DMSO was used for the vehicle control. During the culture, medium were changed every day; and when BIO administration was switched to DAPT, medium were simply removed without additional washing steps.

### Immunohistochemistry

Cochleae were fixed with 4% (wt/vol) paraformaldehyde (Sigma) for 20 minutes. To detect cell proliferation, cochleae were permeabilized in PBS containing 1% Triton X-100 (1% PBS-T) for 15 minutes and labeled with the Click-iT EdU Imaging Kits (Life Technologies) according to the manufacturer's protocol. All cochleae were blocked with 1% PBS-T with 10% goat serum for 1 hour at room temperature. All antibodies were diluted in 1% PBS-T. Primary antibodies used in this study were rabbit anti-MyosinVIIa (1:800 dilution, Proteus BioSciences), goat anti-Sox2 (1:300 dilution, Santa Cruz), chicken anti-EGFP (1:1000 Abcam), and goat anti-Prestin (1:1000 dilution, Santa Cruz). Appropriate Alexa-conjugated secondary antibodies were used for detection. Nuclei were labeled with DAPI (1:1000 dilution, Sigma).

### RNA extraction and real-time PCR

Total RNA isolation was carried out using Trizol (Life Technologies), and mRNA was reverse transcribed with the GoScript^TM^ Reverse Transcription System (Promega) following the manufacturer's protocol. Primer pairs were designed using the online Primer3 software (http://frodo.wi.mit.edu/primer3/, Supplementary Table S11). Quantitative RT-PCR was performed using GoTaq^®^ qPCR Master Mix (Promega) on an Applied Biosystem 7500. The ΔΔCT method with β-actin as the endogenous reference was used to analyze gene expression.

### Cell counting

Fluorescent Z-stack images were collected using a Leica TCS SP8 scanning confocal microscope. For cell counting, Z-projection was performed to project multiple slides of a Z-stack image to a single layer, and the number of Atoh1-eGFP^+^, EdU^+^/eGFP^+^, EdU^+^/Sox2^+^ cells, and EdU^+^/Myo7a^+^ cells were counted in the apical, middle, and basal turns of the cochlea per 100 μm using ImageJ software.

### Statistics

Results were expressed as mean ± SEM, and the statistical analyses were carried out in GraphPad Prism 6.0. Statistical significance was determined with unpaired Student's *t*-tests, and *p* < 0.05 was considered significant.

Further details about cochlear explant cultures, immunohistochemistry, RNA extraction, real-time PCR (primer sequences are listed in Supplemental Experimental Procedures with Table), and cell counting are in the Supplemental Experimental Procedures.

### Supplementary data

Supplementary data includes primer sequences for genotyping and qRT-PCR and 6 tables related to Figures 1–7.

## SUPPLEMENTARY MATERIAL FIGURES AND TABLES


