# Supplementary material for: Wnt activation followed by Notch inhibition promotes mitotic hair cell regeneration in the postnatal mouse cochlea
Source: Oncotarget. 2016 Aug 22;7(41):66754–68. doi: 10.18632/oncotarget.11479 (PMC5341835; doi:10.18632/oncotarget.11479)
Supplement: Supplementary file 1 [file oncotarget-07-66754-s001.pdf]

Wnt activation followed by Notch inhibition promotes mitotic hair cell regeneration in the postnatal mouse cochlea

Supplementary Materials

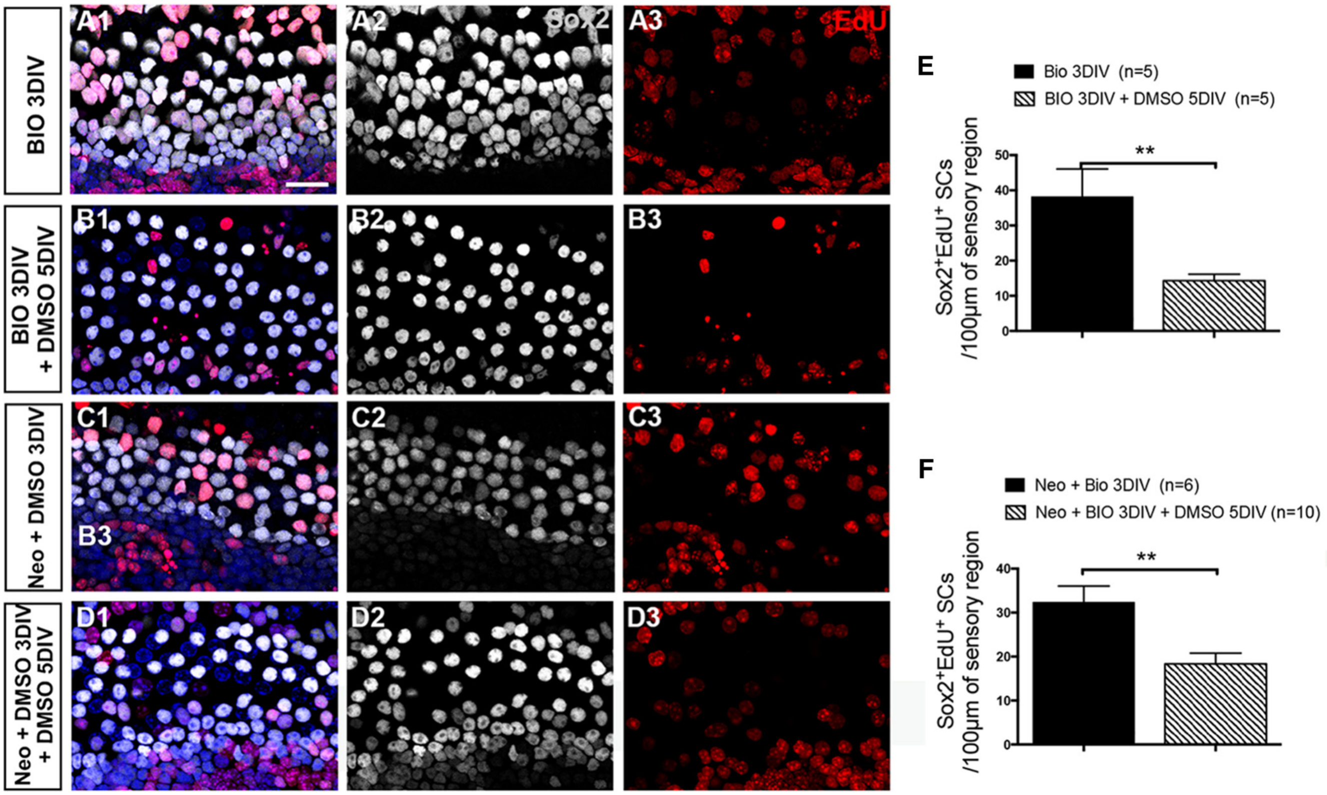

Supplementary Figure S1: The number of proliferative SCs significant decreased after Wnt activation with time prolonging. (**A**–**B**) Images were taken from the apex of the uninjured cochleae. SCs were marked by Sox2 (gray, A2–B2), and proliferating cells were marked by EdU (red, A3–B3). DAPI is blue and the scale bars are 20 μm. (**C**–**D**) Images were taken from the apex of the injured cochleae. SCs were marked by Sox2 (gray, C2–D2), and proliferating cells were marked by EdU (red, C3–D3). DAPI is blue and the scale bars share the same with that in A1. (**E**–**F**) The number of Sox2+/EdU+ cells per 100 μm in the sensory region of the uninjured (E) or injured (F) cochleae. Data are represented as mean ± SEM; $**p < 0.01$, unpaired Student's *t*-tests (two-tailed), see also Table S7.

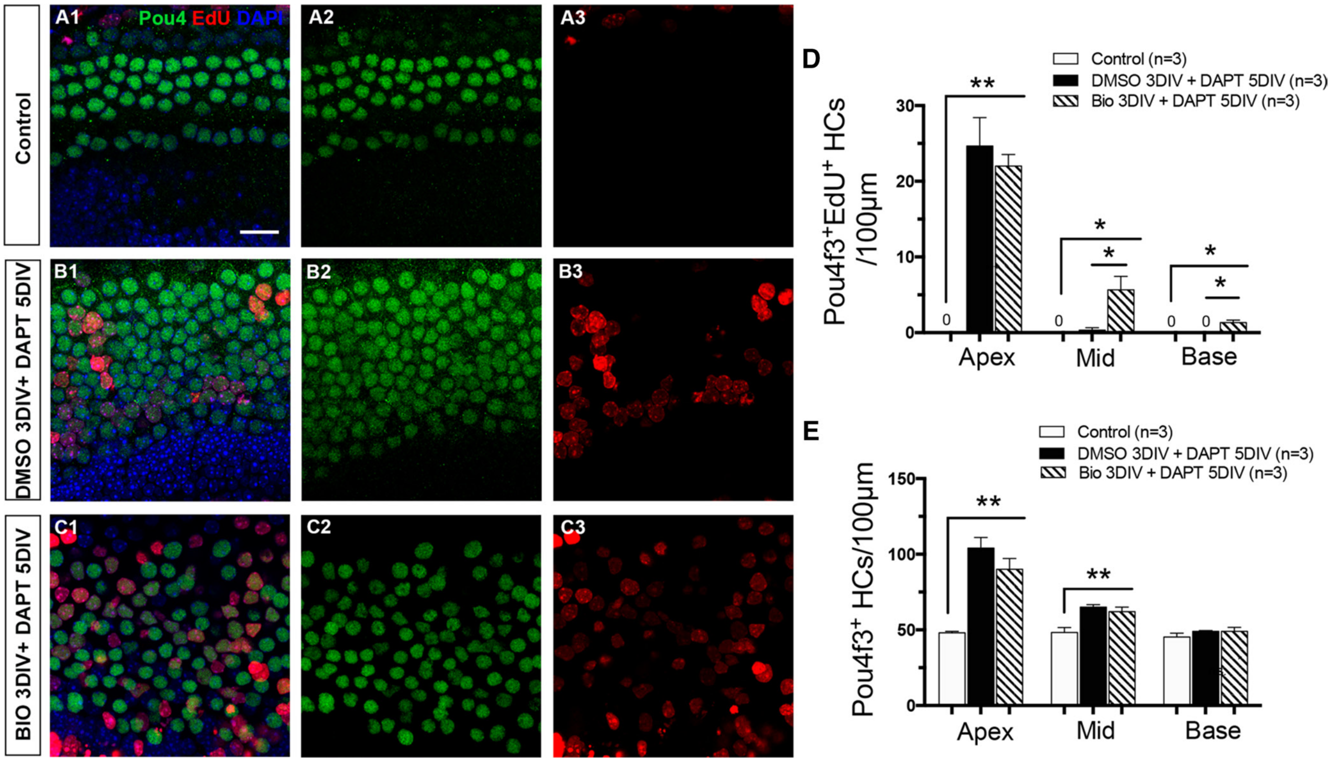

Supplementary Figure S2: Wnt/β-catenin signaling activation followed by Notch signaling inhibition increased mitotic HC regeneration in neonatal mouse cochleae. (**A**–**C**) All images are from the apex of the uninjured cochleae. HCs were marked by Pou4f3 (green, A2–C2), EdU is emplolyed to show mitotic hair cells (red, A3–C3). The scale bar is 20 μm. (**D**) The number of Pou4f3^{+}/EdU^{+} cells per 100 μm from the apex to the base of normal cochleae. (**E**) The number of Pou4f3^{+} HCs per 100 μm from the apex to the base of normal cochleae. Data in D & E are represented as mean ± SEM. *$p < 0.05$, **$p < 0.01$, unpaired Student's *t*-tests (two-tailed), See also Table S8.

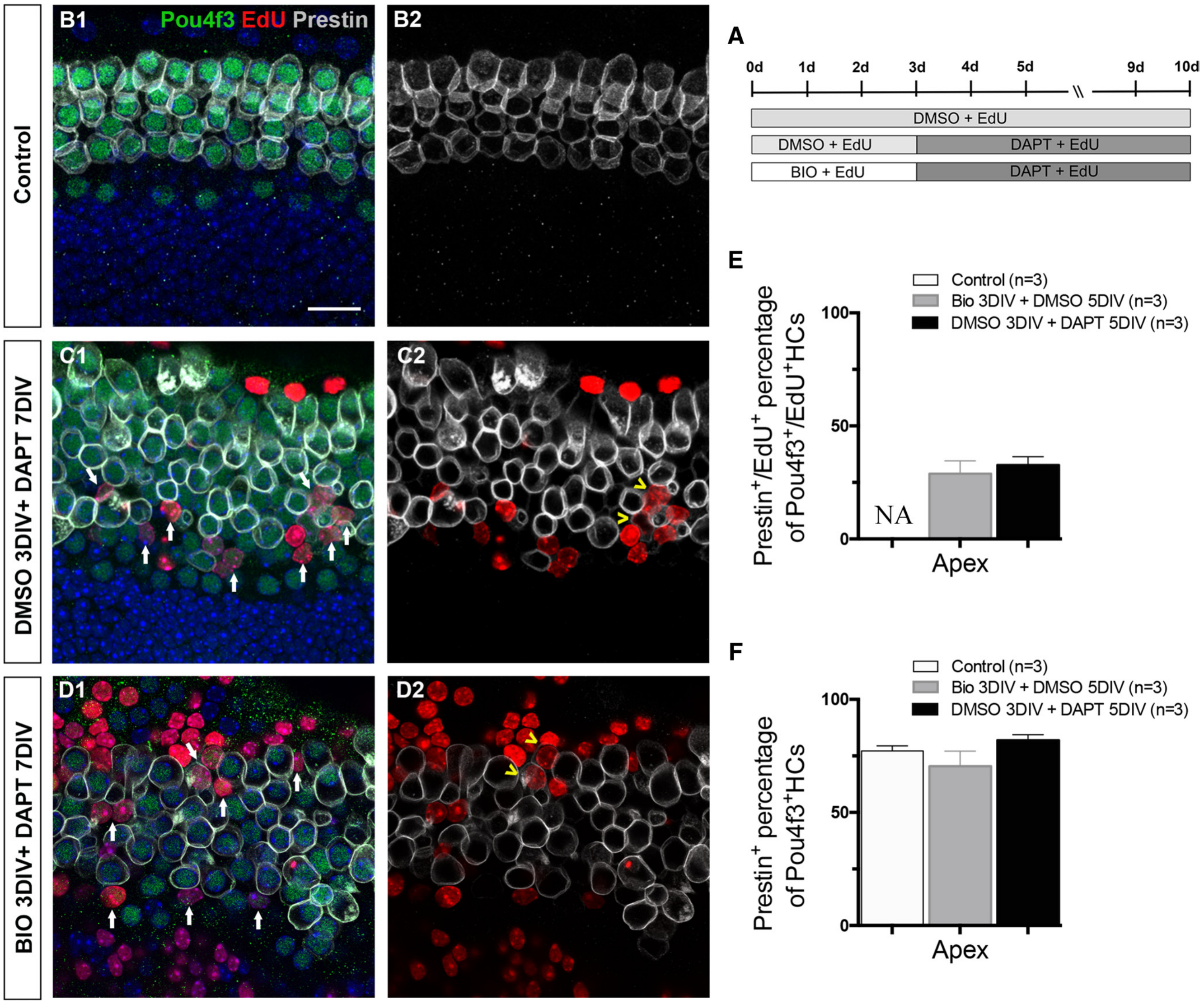

Supplementary Figure S3: The maturation of mitotically regenerated HCs. (**A**) Cochleae of C57/BL mice were dissected at P1 for further treatment. In the trial group, 50 μM DAPT (a γ-secretase inhibitor) was added to the culture media for 7 days after 3 days of BIO treatment. BIO were replaced with DMSO in the controls. (**B**–**D**) All images are from the apex of the uninjured cochleae. HCs were marked by Pou4f3 (green) and Prestin (grey), EdU is emplolyed to show mitotic hair cells (red). White arrows show the $Pou4f3^{+}/EdU^{+}$ cells and yellow arrowheads show the $Prestin^{+}/EdU^{+}$ cells. DAPI is blue and the scale bar is 20 μm. (**E**) The percentage of $Prestin^{+}$cells in $Pou4f3^{+}/EdU^{+}$ cells in the apex of normal cochleae. (**F**) The percentage of $Prestin^{+}$cells in $Pou4f3^{+}$ cells in the apex of normal cochleae. Data in E&F are represented as mean ± SEM, unpaired Student's *t*-tests (two-tailed), See also Table S9.

Supplementary Table S1: Cell counting data for Figure 1

	Sox2+/EdU+ SCs (no damage)		Sox2+/EdU+ SCs (damage)	
	**Control (*n* = 3)**	**BIO 3DIV (*n* = 5)**	**Control (*n* = 3)**	**BIO 3DIV (*n* = 6)**
Apex	0.00 ± 0.00	38.16 ± 3.54	0.00 ± 0.00	32.37 ± 1.51
Mid	0.00 ± 0.00	24.00 ± 2.65	0.00 ± 0.00	13.50 ± 1.06
Base	0.00 ± 0.00	1.33 ± 0.33	0.00 ± 0.00	1.50 ± 0.85

"*n*" is the number of cochleae. "DIV" is days *in vitro*. Data were represented as Mean ± SEM.

Supplementary Table S2: Cell counting data for Figure 2

		**Control (*n* = 3)**	**BIO 3DIV + DMSO 5 DIV (*n* = 3)**	**DMSO 3DIV + DAPT 5DIV (*n* = 3)**	**BIO 3DIV + DAPT 5DIV (*n* = 3)**
eGFP+/EdU+ HCs	Apex	0.00 ± 0.00	2.67 ± 1.33	20.67 ± 1.20	18.67 ± 4.49
	Mid	0.00 ± 0.00	0.67 ± 0.33	0.67 ± 0.67	7.00 ± 1..00
	Base	0.00 ± 0.00	0.33 ± 0.33	0.00 ± 0.00	3.33 ± 0.67
eGFP+ HCs	Apex	48.67 ± 1.20	50.00 ± 3.51	102.00 ± 9.54	88.00 ± 7.02
	Mid	48.33 ± 1.45	49.33 ± 1.86	55.33 ± 1.86	64.00 ± 2.31
	Base	46.67 ± 1.86	43.67 ± 3.18	42.00 ± 1.73	46.33 ± 2.33
The ratio of EdU+ cells to eGFP+ cells (%)	Apex	0.00 ± 0.00	1.96 ± 1.96	20.63 ± 2.30	21.35 ± 5.51
	Mid	0.00 ± 0.00	0.63 ± 0.63	1.13 ± 1.13	10.87 ± 1.20
	Base	0.00 ± 0.00	0.88 ± 0.88	0.00 ± 0.00	7.26 ± 1.56

"*n*" is the number of cochleae. "DIV" is days *in vitro*. Data were represented as Mean ± SEM.

Supplementary Table S3: Cell counting data for Figure 3

	**Control (*n* = 3)**	**BIO 3DIV + DMSO 5 DIV (*n* = 3)**	**DMSO 3DIV +DAPT 5DIV (*n* = 3)**	**BIO 3DIV + DAPT 5DIV (*n* = 3)**
The ratio of Myo7a+ cells to eGFP+ cells (%)	100.00 ± 0.00	98.08 ± 1.03	94.58 ± 0.94	98.30 ± 1.06
The ratio of Myo7a+ cells to eGFP+/EdU+ cells (%)	NA	33.33 ± 16.67	68.72 ± 7.29	95.67 ± 2.27

"*n*" is the number of cochleae. "DIV" is days *in vitro*. Data were represented as Mean ± SEM.

Supplementary Table S4: Cell counting data for Figure 4

		Control (*n* = 5)	BIO 3DIV + DMSO 5DIV (*n* = 5)	DMSO 3DIV + DAPT 5DIV (*n* = 5)	BIO 3DIV + DAPT 5DIV (*n* = 7)
eGFP+/EdU+ HCs	Apex	0.00 ± 0.00	4.40 ± 1.29	14.67 ± 1.80	26.50 ± 8.18
	Mid	0.00 ± 0.00	1.60 ± 0.68	1.00 ± 0.52	10.00 ± 1.86
	Base	0.00 ± 0.00	0.60 ± 0.40	0.17 ± 0.17	3.50 ± 1.19
eGFP+ HCs	Apex	47.80 ± 2.54	45.80 ± 2.46	92.40 ± 6.07	75.57 ± 7.76
	Mid	17.80 ± 3.85	24.40 ± 3.37	35.40 ± 1.75	53.14 ± 2.37
	Base	3.20 ± 0.58	4.60 ± 1.08	9.75 ± 2.59	21.25 ± 2.02
The ratio of EdU+ cells to eGFP+ cells (%)	Apex	0.00 ± 0.00	9.49 ± 2.62	17.14 ± 1.59	27.52 ± 6.89
	Mid	0.00 ± 0.00	7.36 ± 3.66	3.75 ± 1.86	18.85 ± 3.36
	Base	0.00 ± 0.00	5.83 ± 3.63	1.18 ± 1.18	15.76 ± 7.12

"*n*" is the number of cochleae. "DIV" is days *in vitro*. Data were represented as Mean ± SEM.

Supplementary Table S5: Cell counting data for Figure 5

		Control	DMSO 3DIV + DAPT 5DIV	BIO 3DIV + DAPT 5DIV
No damage (*n* = 3)	Sox2+/EdU+ SCs	0	12.00 ± 2.52	23.67 ± 3.71
	Sox2+ SCs	71.00 ± 4.00	25.33 ± 2.91	43.67 ± 5.78
	SC/HC ratio	1.48 ± 0.05	0.19 ± 0.02	0.51 ± 0.11
Damage (*n* = 4)	Sox2+/EdU+ SCs	0	12.25 ± 1.93	25.00 ± 1.68
	Sox2+ SCs	71.00 ± 3.03	28.75 ± 3.30	47.50 ± 1.56
	SC/HC ratio	1.25 ± 0.04	0.30 ± 0.02	0.51 ± 0.04

"*n*" is the number of cochleae. "DIV" is days *in vitro*. Data were represented as Mean ± SEM.

Supplementary Table S6: Cell counting data for Figures 6 and 7

	The composition of EdU+ cells no damage (%)		The composition of EdU+ cells damage (%)	
	Sox2 + (*n* = 4)	Myo7a + (*n* = 4)	Sox2 + (*n* = 5)	Myo7a+ (*n* = 3)
Tom-	24.78 ± 4.23	18.10 ± 3.90	6.74 ± 1.57	8.53 ± 4.66
Tom+	75.22 ± 4.23	81.90 ± 3.90	93.26 ± 1.57	91.47 ± 4.66

"*n*" is the number of cochleae. "DIV" is days *in vitro*. Data were represented as Mean ± SEM.

Supplementary Table S7: Cell counting data for FigureS1

	Sox2$^+$/EdU$^+$ SCs (no damage)		Sox2$^+$/EdU$^+$ SCs (damage)	
	**BIO 3DIV (*n* = 5)**	**BIO 3DIV+ DMSO 5 DIV (*n* = 5)**	**BIO 3DIV (*n* = 6)**	**BIO 3DIV+ DMSO 5 DIV (*n* = 10)**
Apex	38.16 ± 3.54	14.32 ± 1.81	32.37 ± 1.51	18.34 ± 2.44

"*n*" is the number of cochleae. "DIV" is days *in vitro*. Data were represented as Mean ± SEM.

Supplementary Table S8: Cell counting data for Figure S2

		**Control (*n* = 3)**	**DMSO 3DIV + DAPT 5DIV (*n* = 3)**	**BIO 3DIV + DAPT 5DIV (*n* = 3)**
Pou4f3+/EdU+ HCs/100 µm	Apex	0.00 ± 0.00	24.67 ± 3.76	22.00 ± 1.53
	Mid	0.00 ± 0.00	0.33 ± 0.33	5.67 ± 1.76
	Base	0.00 ± 0.00	0.00 ± 0.00	1.33 ± 0.33
Pou4f3+ HCs /100 µm	Apex	48.00 ± 0.58	104.00 ± 7.10	90.00 ± 4.16
	Mid	48.33 ± 1.86	65.00 ± 1.73	62.00 ± 1.73
	Base	45.33 ± 1.45	49.00 ± 0.58	49.00 ± 1.53

"*n*" is the number of cochleae**.** "DIV" is days *in vitro*. Data were represented as Mean ± SEM.

Supplementary Table S9: Cell counting data for Figure S3.

	**Control**	**DMSO 3DIV + DAPT 7DIV (*n* = 3)**	**BIO 3DIV + DAPT 7DIV (*n* = 3)**
The ratio of Prestin$^+$ cells to Pou4f3$^+$/EdU$^+$ HCs (%)	0.00 ± 0.00	28.89 ± 3.24	30.71 ± 1.73
The ratio of Prestin$^+$ cells to Pou4f3$^+$ HCs (%)	77.21 ± 1.31	70.43 ± 3.87	81.92 ± 2.46

"*n*" is the number of cochleae. "DIV" is days *in vitro*. Data were represented as Mean ± SEM.

Supplementary Table S10: Primers for genotyping

Gene		Forward Primer	Reverse Primer
Atoh1 (eGFP)	WT	/	/
	Mu	5′-CACATGAAGCAGCACGACTT-3′	TGCTCAGGTAGTGGTTGTCG
Lgr5	WT	5′-CTGCTCTCTGCTCCCAGTCT-3′	5′-ATACCCCATCCCTTT TGAGC-3′
	Mu	5′-CTGCTCTCTGCTCCCAGTCT-3′	5′-GAACTTCAGGGTCAG CTTGC-3′
tdTomato	WT	5′-AAGGGAGCTGCAGTGGAGTA-3′	5′-CCGAAAATCTGTGGG AAGTC-3′
	Mu	5′-GGCATTAAAGCAGCGTATCC-3′	5′-CTGTTCCTGTACGG CATGG-3′

Supplementary Table S11: Primers for for qRT-PCR

Gene	Forward Primer	Reverse Primer
$p27^{kip1}$	5′-CGGTGCCTTTAATTGGGTCT-3′	5′-AGCAGGTCGCTTCC TCATC-3′
β-catenin	5′-ATGCGCTCCCCTCAGATGGTGTC-3′	5′-TCGCGGTGGTGAGA AAGGTTGTGC-3′
Axin2	5′-TGACTCTCCTTCCAGATCCCA-3′	5′-TGCCCACACTAGG CTGACA-3′
Sp5	5′-CGGACCTGGGCAAGCACT-3′	5′-GGGTGGAAAAGTCTG GAGGG-3′
Lgr5	5′-CCTACTCGAAGACTTACCCAGT-3′	5′-GCATTGGGGTGAAT GATAGCA-3′
Hes1	5′-ACGACACCGGACAAACCA-3′	5′-ATGCCGGGAGCT ATCTTTCT-3′
Hes5	5′-TGCTCAGTCCCAAGGAGAAA-3′	5′-AGCTTGGAGTTGG GCTGGT-3′
Hey1	5′-CACTGCAGGAGGGAAAGGTTAT-3′	5′-CCCCAAACTCC GATAGTCCAT-3′
Notch1	5′-GGAGGACCTCATCAACTCACA-3′	5′-CGTTCTTCAGGAG CACAACA-3′
Jag1	5′-TGTGCAAACATCACTTTCACCTTT-3′	5′-GCAAATGTGTTCG GTGGTAAGAC-3′
β-actin	5′-GGCTGTATTCCCCTCCATCG-3′	5′-CCAGTTGGTAACAA TGCCATGT-3′
